# Surface-Modified Carboxylated Cellulose Nanofiber Hydrogels for Prolonged Release of Polyhexamethylene Biguanide Hydrochloride (PHMB) for Antimicrobial Applications

**DOI:** 10.3390/polym15173572

**Published:** 2023-08-28

**Authors:** Pichapar O-chongpian, Tanpong Chaiwarit, Kittisak Jantanasakulwong, Pornchai Rachtanapun, Patnarin Worajittiphon, Nutthapong Kantrong, Pensak Jantrawut

**Affiliations:** 1 Department of Pharmaceutical Sciences, Faculty of Pharmacy, Chiang Mai University, Chiang Mai 50200, Thailand; pichaparo@gmail.com (P.O.-c.); tanpong.c@gmail.com (T.C.); 2 Division of Packaging Technology, Faculty of Agro-Industry, Chiang Mai University, Chiang Mai 50100, Thailand; kittisak.jan@cmu.ac.th (K.J.); pornchai.r@cmu.ac.th (P.R.); 3 Center of Excellence in Agro Bio-Circular-Green Industry (Agro BCG), Chiang Mai University, Chiang Mai 50200, Thailand; 4 Department of Chemistry, Faculty of Science, Chiang Mai University, Chiang Mai 50200, Thailand; patnarin.w@cmu.ac.th; 5 School of Dentistry, Mae Fah Luang University, Chiang Rai 57100, Thailand; nutthapong.kan@mfu.ac.th

**Keywords:** carboxylated cellulose nanofibers, hydrogel, TEMPO oxidization, polyhexamethylene biguanide hydrochloride, antimicrobial applications

## Abstract

The surface modification of cellulose nanofibers (CNFs) using a 2,2,6,6-tetramethylpiperidine-1-oxyl (TEMPO)/sodium bromide (NaBr)/sodium hypochlorite (NaClO) system was successful in improving their hydrophilicity. Following that, we fabricated hydrogels containing carboxylated cellulose nanofibers (c-CNFs) and loaded them with polyhexamethylene biguanide (PHMB) using a physical crosslinking method, aiming for efficient antimicrobial uses. The morphological and physicochemical properties of all hydrogel formulations were characterized, and the results revealed that the 7% c-CNFs-2 h loaded with PHMB formulation exhibited desirable characteristics such as regular shape, high porosity, good mechanical properties, suitable gel content, and a good maximum swelling degree. The successful integration of PHMB into the c-CNF matrix was confirmed by FTIR analysis. Furthermore, the 7% c-CNFs-2 h loaded with the PHMB formulation demonstrated PHMB contents exceeding 80% and exhibited a prolonged drug release pattern for up to 3 days. Moreover, this formulation displayed antibacterial activity against *S. aureus* and *P. aeruginosa.* In conclusion, the novel approach of c-CNF hydrogels loaded with PHMB through physical crosslinking shows promise as a potential system for prolonged drug release in topical drug delivery while also exhibiting excellent antibacterial activity.

## 1. Introduction

Nanocellulose, which can be extracted from cellulose sources such as plants, algae, fungi, and bacteria, is a fiber with a diameter of less than 100 nm and a length in the micrometer range [1,2]. It possesses numerous hydroxyl groups and exhibits high mechanical strength. Additionally, nanocellulose has important properties, including high flexibility, biocompatibility, non-toxicity, biodegradability, renewability, and low cost [3]. For these reasons, nanocellulose has received a lot of attention and interest from researchers as a suitable nanostructure for developing new high-value materials in various fields. Cellulose nanofibers (CNFs), which consist of both amorphous and crystalline cellulose domains, are micrometer-long entangled fibrils [4]. CNFs have many unique properties, such as biodegradability, biocompatibility, high strength, good mechanical properties, a large specific surface area, and the ability to form a strong entangled nanoporous network with an abundant pore structure. Additionally, CNFs demonstrate water swelling and absorptivity characteristics [5]. Furthermore, their nanostructure clearly illustrates their advantages as drug carriers, including their high drug loading rate and capacity for long-term sustained release [6]. Li X. et al. fabricated a cellulose nanomesh (CNM) using a mild acid vapor with a dilute enzymatic hydrolysis preparation method. Their study revealed that CNMs exhibit many benefits, such as a large specific surface area, a high water retention capacity, a high drug loading rate, and a sustained-release effect due to their gel matrix network [6]. CNFs have the potential to be combined with other polymers, enabling the creation of nanocellulose hydrogels for various applications, such as wound dressings, food, cosmetics, tissue engineering, and bioprinting [7,8,9,10].

In our previous study, we developed composite nanocellulose fiber-based hydrogels loaded with clindamycin hydrochloride with calcium ion and citric acid as crosslinking agents. The mass ratio between the CNFs and the two polymeric precursors (low methoxy pectin and sodium alginate) was adjusted to create the CNF-based hydrogels. However, the dispersion of the CNFs in aqueous media remained a significant challenge. Thus, we incorporated polyethylene glycol (PEG) into the CNFs to enhance their dispersity in the aqueous phase [11]. It is worth noting that using CNFs alone for hydrogel fabrication can be challenging due to their weak macroscopic structure. Nevertheless, CNFs with functional groups such as carboxyl and amino groups can be utilized to create hydrogels with higher porosity and water capacity [12]. Moreover, Liu Y. et al. fabricated novel physically crosslinked composite 2,2,6,6-tetramethylpiperidine-1-oxyl (TEMPO)-CNFs and polydopamine hydrogels through ion crosslinking with calcium ions as a crosslinker loaded with tetracycline hydrochloride. The TEMPO-CNFs provided a 3D framework structure, while polydopamine served as a photothermal reinforcing agent and drug carrier. Their results indicated that the polydopamine/TEMPO-CNF composite hydrogel exhibited easy preparation, suitable mechanical properties, excellent control over drug delivery, and enhanced wound healing abilities [12]. Furthermore, Liu R. et al. prepared a nanocomposite hydrogel from carboxylated CNFs (CNF) and gelatin (G) loaded with aminated silver nanoparticles (Ag-NH_2_ NPs) for antibacterial purposes and wound healing. They observed that the crosslinking reactions involved ionic interactions between the amino group (-NH_2_) and the carboxyl group (-COOH) of the components. They concluded that the CNF/G/0.5 mg/mL of the Ag-NH_2_ NP hydrogel dressing showed improved mechanical performance, excellent biocompatibility, antibacterial activity, and accelerated wound healing [13]. Numerous studies have evidently improved the structure of CNFs using TEMPO oxidation. This technique selectively converts C6 primary hydroxyl groups within the cellulose molecular chain into carboxyl groups, resulting in the negatively charged dispersion of CNFs. This dispersion exhibits notable stability and dispersibility in water, preventing sedimentation and demonstrating ionic thixotropy [14]. Based on the findings of Yuan Z. et al., the utilization of TEMPO oxidation and ultrasonic treatments on algae cellulose retains the high aspect ratio of algae cellulose nanofibers while also improving their water dispersion and stability. This provides opportunities for the creation of ionic gels and ensures the consistent distribution of ion sites in the gel matrix. These findings were confirmed by the application of 3D Raman imaging technology [14].

**Figure 1:**
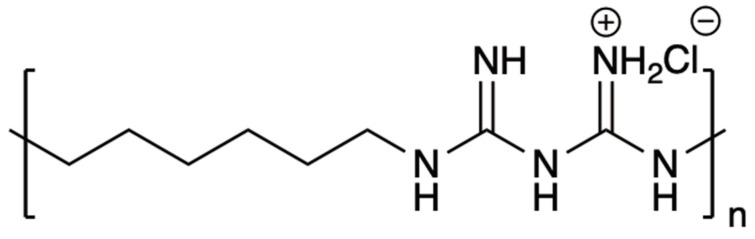
Chemical structure of polyhexamethylene biguanide hydrochloride (PHMB).

Polyhexamethylene biguanide hydrochloride (PHMB) is a biocidal cationic oligomer with an average of 7–11 biguanide groups spaced by flexible hexamethylene segments. It exhibits a highly effective, broad spectrum of activity against microorganisms while having low toxicity to humans [15,16]. The chemical structure of PHMB is shown in Figure 1. It is widely used in clinics, cleaning products, cosmetics, veterinary products, and various industries [16,17,18]. The mechanism of action of PHMB involves multiple pathways. Its interaction with negatively charged phospholipids disrupts the stability of cells’ phospholipid bilayer and leads to leakage, potentially resulting in cell death. Additionally, the entry of PHMB into bacterial cells may occur by mechanisms similar to those proposed for cationic peptides. Upon entering the cells, PHMB’s interaction with the DNA backbone can play a significant role in cell death by blocking the DNA replication pathway and inhibiting DNA repair processes [16]. PHMB demonstrates effectiveness against bacteria commonly associated with skin infections, including *Pseudomonas aeruginosa*, *Streptococcus pyogenes*, and *Staphylococcus aureus*. Commercial wound dressings that release PHMB are currently available. These dressings utilize matrices made from a wide range of polymers, including natural polymers such as cotton, viscose, rayon, bacterial cellulose, and extracellular matrix biopolymers, as well as synthetic polymers such as polyesters and polyurethanes [19]. Eberlein et al. conducted a study that compared the treatment of wounds with a PHMB-containing bacterial cellulose dressing (Suprasorb X+PHMB) to the local standard of silver dressings in terms of pain relief. Their results revealed that Suprasorb X+PHMB was significantly more effective and efficient in eliminating the critical bacterial load [20]. Jin J. et al. used gelatin and glycerin with glutaraldehyde as a crosslinking agent to create a PHMB hydrogel-modified wound scaffold dressing with antibacterial activity [21]. Additionally, Napavichayanun et al. created a biocellulose wound dressing using silk sericin and bacterial cellulose loaded with PHMB. They observed that the dressing exhibited antibacterial properties and promoted collagen synthesis during the healing process. In order to effectively eliminate all tested bacteria (*B. subtilis*, *S. aureus*, MRSA, *E. coli*, *A. baumannii*, and *P. aeruginosa*), a minimum loading concentration of 0.3% *w*/*v* of PHMB was required [22].

Consequently, this study aimed to improve the dispersion of CNFs through surface modification using TEMPO oxidation. We investigated the effect of the oxidation time on the quality parameters of TEMPO-oxidized CNFs, including the degree of oxidation, carboxylic acid content, and yield percentage. The structures of c-CNFs were characterized using a field emission scanning electron microscope (FESEM), Fourier transform infrared (FTIR) spectroscopy, and X-ray diffraction (XRD) analysis. The optimal formulation for surface-modified CNF hydrogels was investigated. The morphology, mechanical properties, drug loading content, in vitro drug release profile, and antibacterial effects of PHMB on *Staphylococcus aureus* and *Pseudomonas aeruginosa* were determined. Hypothetically, the surface modification of CNFs should improve their dispersion in aqueous media and enable the development of surface-modified CNF hydrogels loaded with PHMB through physical crosslinking, eliminating the need for other chemical crosslinking agents for antimicrobial applications.

## 2. Materials and Methods

### 2.1. Materials

Cellulose nanofibers (CNFs, a white dry powder with a nominal fiber width of 50 nm) were purchased from CelluloseLab, New Brunswick, Canada. 2,2,6,6-Tetramethylpiperidine-1-oxyl (TEMPO 99.6%) was purchased from Sigma-Aldrich, Missouri, USA. Sodium bromide (NaBr), sodium hypochlorite (NaClO), and sodium hydroxide (NaOH) were purchased from Merck KGaA, Darmstadt, Germany. Polyhexamethylene biguanide (PHMB) was purchased from Carbosynth Ltd., Compton, UK. Distilled water was used as the solvent for preparing carboxylated cellulose nanofibers and hydrogels. All the other reagents were analytical grade.

### 2.2. Preparation of Carboxylated Cellulose Nanofibers

Carboxylated cellulose nanofibers (c-CNFs) were synthesized via TEMPO-mediated oxidation. Initially, a dispersion of 2 g of unmodified CNFs (u-CNFs) in 270 mL of deionized water was prepared. The u-CNF dispersion was combined with a solution of NaBr (206 mg) and TEMPO (31.2 mg) in 20 mL of deionized water. The reaction was initiated by adding 27 mL of 10% (*w*/*w*) NaClO, while maintaining the pH at 10.3 through the incremental addition of 0.5 M NaOH. Stirring the mixture at room temperature (25 ± 2 °C) was sustained for durations of 1, 2, and 3 h. To terminate the reaction, 1% (*v*/*v*) ethanol was introduced, and subsequent centrifugation (8000 rpm for 30 min) and resuspension steps were performed. Centrifugation continued until the conductivity of the purification water reached <0.005 mS/cm. The purified c-CNF suspension was freeze-dried (Christ Beta 2–8 LD-plus, Osterode am Harz, Germany) for 24 h [23]. The degree of oxidation and the carboxyl group content of the c-CNF suspension were determined by conductometric titration [24].

### 2.3. Yield Measurement

The yield percentage of carboxylated CNFs (c-CNFs) was measured using the gravimetric analysis method. The percentage yield of nanocellulose was calculated using the following Equation (1).

Yield (%) = (W_c-CNFs_/W_u-CNFs_) × 100% (1)

where W_c-CNFs_ is the weight of the dry carboxylated CNFs (g) and W_u-CNFs_ is the dry weight of the initial unmodified CNFs (g).

### 2.4. Morphological Characterizations of u-CNFs and c-CNFs

The morphology of the u-CNFs and c-CNFs was examined using a field emission scanning electron microscope (FESEM) (TESCAN CLARA, Brno-Kohoutovice, Czech Republic). Before imaging, the specimens were mounted onto aluminum stubs using double-sided carbon tape (NEM tape, Nisshin Co., Ltd., Tokyo, Japan) and subsequently platinum-coated for 2 min. These prepared samples were then placed on the imaging stage of the device. FESEM images of all CNFs were obtained using a secondary electron detector operating at an acceleration voltage of 15 keV in high vacuum mode with magnifications set at ×2000.

### 2.5. Fourier Transform Infrared Spectroscopy (FTIR)

Unmodified CNFs (u-CNFs) and carboxylated CNFs (c-CNFs) were identified and evaluated for their functional groups by Fourier transform infrared spectroscopy (FTIR) using a Nicolet iS10 FTIR spectrometer (Thermo Acientific Inc., Waltham, MA, USA). Briefly, a powder of u-CNFs and c-CNFs (1 mg) was placed on an FTIR plate and inserted into the instrument for reading. Absorbance levels were measured at a resolution of 4 cm^−1^ in transmittance mode from 400 to 4000 cm^−1^.

### 2.6. Determination of the Degree of Oxidation and the Carboxylic acid Content of Carboxylated Cellulose Nanofibers

The degree of oxidation (DO) and carboxylic acid content (CC) of c-CNFs were determined by conductometric titrations. Initially, 50 mg of c-CNFs was dispersed in 15 mL of 0.01 M HCl and mixed for 10 min to exchange the Na cations from the COO^−^ group for H^+^. The indicator phenolphthalein (0.1%) was added to the mixture. The suspensions were titrated after that by adding 0.01 M NaOH solution in 0.1 mL increments while monitoring the conductivity of the suspensions. The curve demonstrated the presence of a strong acid (excess of HCl) and a weak acid (carboxylic acid). Finally, the degree of oxidation and the carboxylic acid content were calculated using Equations (2) and (3) [24,25].

Degree of oxidation (DO) = 162 × (V_2_ − V_1_) × C/[w − (36 × C) × (V_2_ − V_1_)] (2)

Carboxylic acid content (CC, mmol/g) = C × (V_2_ − V_1_)/w (3)

where, V_1_ and V_2_ are the equivalent volumes of the added NaOH in liters, C is the concentration of the NaOH (0.01 M), and w is the dry weight of the sample in grams.

### 2.7. X-ray Diffraction (XRD) Analysis of u-CNFs and c-CNFs

XRD spectra for u-CNFs and c-CNFs were obtained using an X-ray diffractometer (Miniflex ll, Rigaku Corporation, Tokyo, Japan) operating at 30 kV and 20 mA with CuKα radiation. The measurements were conducted in the 2θ range of 3–80° with a step interval of 0.02°. The crystallinity index (CrI) was calculated using Equation (4) based on the highest intensity peak (I_200_) and the intensity minimum (I_am_) [26].

Crystallinity index (Crl) = [(I_200_ − I_am_)/I_200_] × 100% (4)

where I_200_ is the XRD intensity of the crystal plane (200) and I_am_ is the XRD intensity of the amorphous region.

### 2.8. Preparation of Carboxylated Cellulose Nanofiber Hydrogels

c-CNF hydrogels were prepared by dispersing different percentages (1–7% *w*/*v*) of c-CNFs in deionized water (DI) using a homogenizer (IKA T25 Ultra-Turrax, IKA Laboratory Technology, Staufen, Germany) under homogenization at 25 °C and 6000 rpm for 5 min. Then, each formulation was transferred into Petri dishes, and 0.3% *w*/*v* PHMB was poured into the CNF dispersion. After 24 h of immersion, the crosslinked hydrogels were removed from the Petri dishes and washed with DI water, and excess surface DI water was gently blotted with filter paper. After that, the hydrogels were freeze-dried (Christ Beta 2–8 LD-plus, Osterode am Harz, Germany) for 24 h to obtain the freeze-dried c-CNF hydrogels.

### 2.9. c-CNF Hydrogel Characterizations

#### 2.9.1. Morphological Characterizations

The morphology of the freeze-dried c-CNFs hydrogels was examined using a field emission scanning electron microscope (FESEM) (TESCAN CLARA, Brno-Kohoutovice, Czech Republic). The procedure was the same as described in Section 2.4. Subsequently, SEM images of all c-CNFs hydrogels were collected using a secondary electron detector at an acceleration voltage of 15 keV under high vacuum mode at magnifications of ×1000.

#### 2.9.2. Fourier Transform Infrared Spectroscopy

Chemical interactions between the c-CNF hydrogel formulations and PHMB were analyzed using an FTIR spectrometer (FTIR-4700, Jasco, Tokyo, Japan). The procedure for analysis was the same as described in Section 2.5.

#### 2.9.3. Mechanical Strength Test

To evaluate the mechanical strength of the selected c-CNF hydrogels, we utilized a texture analyzer, TA.XT plus (Stable Micro Systems, Surrey, UK). The procedures described in [11] were followed. Each hydrogel was assessed five times. After the experiments, we calculated the puncture strength (N/mm^2^) using Equation (5).

Puncture strength = F_max_/A (5)

where Fmax is the force at the breaking point (N), and A is the surface area in contact with the probe’s surface (mm^2^).

#### 2.9.4. Gel Content

The crosslinking efficiency was evaluated by conducting a gel content analysis in phosphate-buffered saline (PBS) with a pH of 7.4, maintained at 37 ± 0.5 °C, as described in [11]. Freeze-dried c-CNF hydrogels were cut into dimensions of 1 cm × 1 cm. The experiment was performed in triplicate under the same conditions, and the average gel content (GC) values were calculated using the following Equation (6).

Gel content (%GC) = W_d_/W_i_ × 100 (6)

#### 2.9.5. Swelling Ratio

The swelling ratio of freeze-dried CNF-based hydrogels was determined through a gravimetric method in 20 mL of PBS with a pH of 7.4 maintained at 37 ± 0.5 °C for 24 h, as described in [11]. Three randomly selected samples were cut into 1 cm × 1 cm. The calculation of the maximum swelling degree (%) was calculated using Equation (7).

Maximum swelling degree (%MSD) = (W_w_ − W_d_)/W_d_ × 100 (7)

### 2.10. PHMB Loading Content

For each formulation of freeze-dried c-CNF hydrogel with PHMB, three random samples were placed into vials containing 20 mL of deionized water. These vials were allowed to stand for 24 h, ensuring the complete dissolution of the hydrogels. The dissolution process was facilitated by a magnetic stirrer set at 100 rpm, maintaining room temperature. After dissolution, the solution samples were filtered using a 0.45 μm membrane filter to exclude minor particles. Subsequently, the filtered samples were subjected to dilution. The average PHMB content was determined using a UV spectrophotometer (UV2600i, Shimadzu Corporation, Kyoto, Japan) operating at a wavelength of 236 nm. The quantification of PHMB content was calculated using a standard PHMB solution with a concentration range from 2.5 μg/mL to 17.5 μg/mL, which displayed high linearity (r^2^ = 0.996). Equation (8) was used to calculate the drug content of the PHMB-incorporated hydrogel.

(8) $$\mathrm{PHMB}\;\mathrm{loading}\;\mathrm{content}\;(\%)=\frac{Amount\;of\;drug\;in\;hydrogel}{Theorectical\;drug}\times 100$$

### 2.11. In Vitro Drug Release Profile

c-CNF hydrogels containing PHMB, shaped as squares measuring 1 cm × 1 cm, were immersed in 20 mL of PBS buffer with a pH of 7.4 at 37 ± 0.5 °C. The medium was continuously stirred using a magnetic bar at 50 rpm. At predetermined intervals (0.5, 1, 2, 3, 6, 12, 24, 36, 48, 72, and 84 h), 2 mL of the dissolution media were collected, and an equal volume of PBS (2 mL) was replaced. The collected samples were analyzed for PHMB release using a UV-Vis spectrophotometer (UV2600i, Shimadzu, Kyoto, Japan) at 236 nm. All dissolution experiments were performed in triplicate. The amount of released drug was calculated using the following Equation (9).

(9) $$\mathrm{Amount}\;\mathrm{of}\;\mathrm{released}\;\mathrm{drug}\;(\%)=\frac{Amount\;of\;released\;drug\;at\;the\;specific\;time}{Amount\;of\;drug\;in\;hydrogel}\times 100$$

### 2.12. Antimicrobial Test

The antibacterial activity of the c-CNF hydrogels was assessed using the disc diffusion technique as described in [27]. All evaluated c-CNF hydrogel samples underwent sterilization under ultraviolet light for 1 h before the antibacterial test. Briefly, c-CNFs-PHMB were applied to prepared bacterial agar plates. As a negative control, c-CNFs without PHMB were utilized, while antibiotic assay discs from Whatman (GE Healthcare, Pittsburgh, PA, USA) loaded with a 0.3% PHMB solution served as the positive control. Each experiment was conducted in triplicate.

### 2.13. Statistical Analysis

The data presented were indicated as mean ± standard deviation (S.D.). Statistical significance was determined through the one-way ANOVA test using SPSS statistics software version 17.0 (IBM Corporation, Armonk, NY, USA). A significance level of *p* < 0.05 was considered indicative of statistically significant differences.

## 3. Results and Discussion

### 3.1. Preparation and Morphological Characteristics of c-CNFs

**Figure 2:**
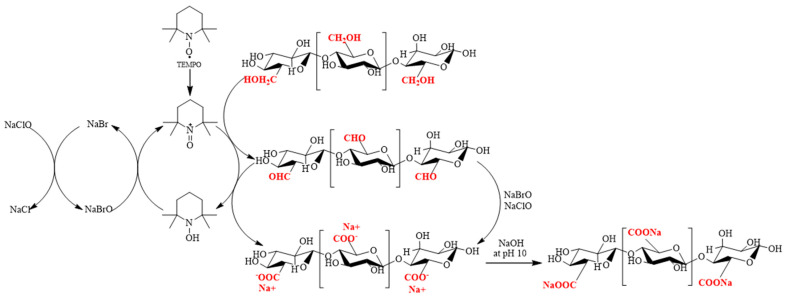
Schematic of the modification cellulose nanofibers by TEMPO−mediated oxidation.

The surface modification of nanocellulose can be achieved by three main methods: imparting ionic charges to nanocellulosic surfaces, generating nanocellulosic materials with hydrophobic surfaces, and polymer grafting on nanocellulose [28,29]. The oxidation method, specifically the method that works by imparting ionic charges, is commonly used to improve the surface properties of nanocellulose using oxidative agents. The most commonly used chemical in the surface modification of nanocellulose is 2,2,6,6-tetramethylpiperidine-1-oxyl (TEMPO), which is a weak oxidizing agent. It can react with sodium hypochlorite (NaClO) and halogen salts such as sodium bromide (NaBr) and sodium chloride (NaCl) in an alkaline state. By oxidation, TEMPO generates a nitrosium ion (+N=O) that contains positively charged nitrogen atoms (a nitrogen carbonyl cation), resulting in a potent oxidizing agent [30]. The oxidation process converts the hydroxyl groups at the carbon position 6 (C6) of the nanocellulose fibers into aldehyde and carboxyl groups, which are then ionized into sodium carboxylates at pH 10, as shown in Figure 2. Increasing the surface charge density of the CNFs allows for the formation of physically crosslinked CNF hydrogels [31].

**Figure 3:**
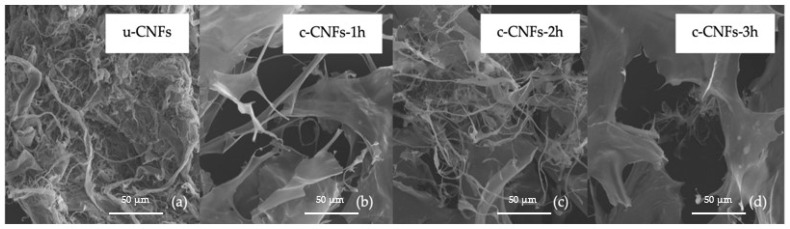
The morphology of the freeze-dried u-CNFs and c-CNFs in the variation of reaction time (1, 2 and 3 h.) by FESEM micrographs at 2000×.

In the present study, the surface modification of CNFs was conducted by oxidation using the TEMPO/NaBr/NaClO system with variations in reaction time at 1, 2, and 3 h. Following modification and freeze-drying, all c-CNFs exhibited a fluffy and uniformly white appearance. The internal morphology of the c-CNFs was examined using FESEM micrographs, as shown in Figure 3. The results demonstrated a significant difference in morphology between the unmodified and modified cellulose nanofibers. The u-CNFs had diameters of 50 nm and lengths in the hundreds of micrometers (Figure 3a), whereas the c-CNFs, after modification with varying reaction times of 1, 2, and 3 h, exhibited shorter lengths and were arranged in thicker ribbons and thin film fragments measuring several micrometers (Figure 3b–d). Consequently, the modified cellulose nanofibers are more prone to internal morphological changes and dispersion in aqueous solutions, indicating that a substantial amount of carboxylic acid on the c-CNF surfaces is crucial for extensive CNF self-assembly or inter-CNF interactions through hydrogen bonding [25].

The yield percentages of c-CNFs obtained through oxidation using the TEMPO/NaBr/NaClO system varied with reaction time (1, 2, and 3 h.) and were measured as 75.46 ± 0.82% *w*/*w*, 60.08 ± 0.73% *w*/*w*, and 51.43 ± 0.45% *w*/*w* of the dry raw material, respectively. When compared to other studies using TEMPO-mediated oxidation, Gond et al. reported a yield percentage of 65% [32]. Additionally, our study’s yield aligned with the results of Xu H. et al., who observed a pulp yield ranging from 40% to 90% during TEMPO-mediated oxidation lasting between 0 and 120 min. These results indicate that the yield decreases with increased reaction time [33]. This decrease can be attributed to the dissolution of water-soluble cellulosic components, which are subsequently removed during the cleaning process [31,33,34].

### 3.2. Fourier Transform Infrared Spectroscopy (FTIR)

**Figure 4:**
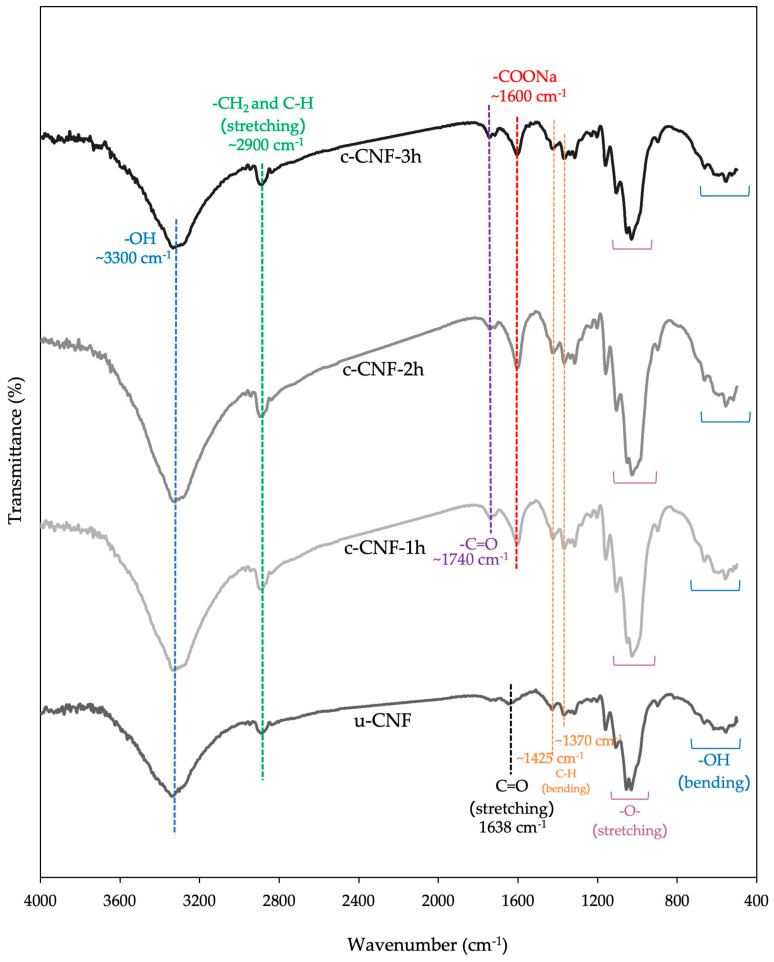
FTIR spectra of the u-CNFs and c-CNFs in the variation of reaction time (1, 2 and 3 h) by TEMPO−mediated oxidation.

FTIR spectroscopy was conducted to evaluate possible interactions among materials by analyzing the chemical shift or intensity change of functional groups, which are related to chemical interactions among substances after oxidation, and to confirm the effectiveness of the synthesis [26]. Figure 4 shows the absorbance bands of representative spectra of unmodified CNFs (u-CNFs) and carboxylated CNFs (c-CNFs) at different times of the modification process. This study demonstrated that the broad and intense band observed at 3200–3600 cm^−1^ corresponds to O-H stretching, while the wide band at 2900 cm^−1^ represents -CH_2_ and C-H stretching. The band around 1630 cm^−1^ corresponds to C=O stretching, and the bands at 1425 cm^−1^ and 1370 cm^−1^ indicate the bending vibrations of C-H [30,31]. The complex bands observed at 895–1200 cm^−1^ represent -O- stretching, and the band at 555–660 cm^−1^ represents O-H bending out of the plane [30,35,36]. The absorbance bands in the spectra of c-CNFs differ from those of u-CNFs. The slight shift in the peak position from 1630 cm^−1^ to 1600 cm^−1^ after TEMPO oxidation is attributed to the stretching of the carboxylate groups (-COO^−^), indicating the successful conversion of the hydroxyl group at the C6 position into a carboxylate group through cellulose oxidation [31,34]. Furthermore, the presence of the carbonyl stretching of COO- of c-CNFs can be observed by the spectrum differences from u-CNFs, with the peak located around 1740 cm^−1^ [30,34]. In a previous study, Tran Thi Thanh Hop et al. prepared TEMPO-oxidized nanocellulose from bleached wood pulps. Additionally, Hassan et al. developed TEMPO-oxidized nanocellulose films derived from coconut residues, such as coconut shells and coconut husks. Both studies found similar characteristic FTIR peaks in TEMPO-oxidized CNFs [26,31].

### 3.3. Degree of Oxidation, Carboxylic Acid Content, and X-ray Diffraction (XRD) Analysis of u-CNFs and c-CNFs

The degree of oxidation (DO) and carboxylic acid content (CC) can be determined by conductometric titrations. Typically, the maximum degree of oxidation of nanocellulose is approximately close to 0.10 [24,37]. Our results indicated that the DO values of c-CNFs-1 h, c-CNFs-2 h, and c-CNFs-3 h were 0.0691, 0.0993, and 0.0971, respectively, as presented in Table 1. This suggests that c-CNFs-2 h and c-CNFs-3 h have undergone oxidation of almost all the surface-accessible hydroxyl groups of nanocellulose into carboxylated groups [24]. Furthermore, our study calculated the CC values of c-CNFs-1 h, c-CNFs-2 h, and c-CNFs-3 h, all of which were approximately in the range of 0.40 to 0.60 mmol/g. The carboxylic content of TEMPO-oxidized CNFs depends on the cellulose source and the reaction conditions [38,39]. However, no significant differences were observed in the DO and CC values between c-CNFs-2 h and c-CNFs-3 h.

**Figure 5:**
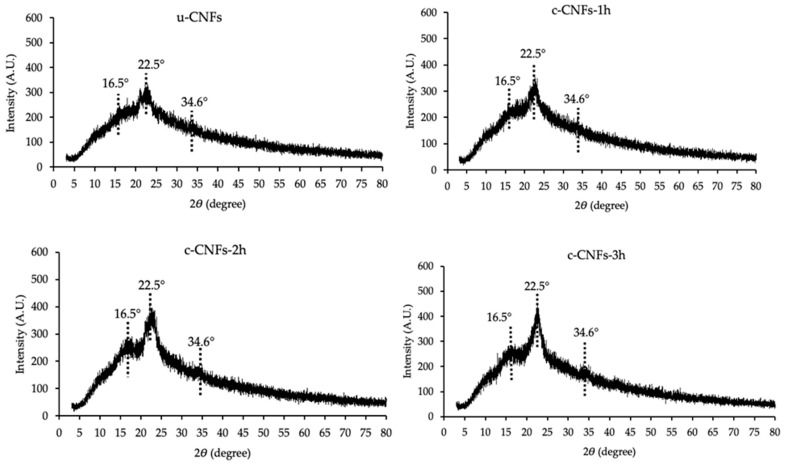
X-ray diffraction of the u-CNFs and c-CNFs with the variation of reaction time (1, 2, and 3 h) for TEMPO-mediated oxidation.

The chemical characteristics and crystallinity index (CrI) of u-CNFs and c-CNFs were investigated by XRD characterization. The crystallinity index of c-CNFs is a critical factor that affects their mechanical strength within the polymer matrix. The XRD analysis demonstrated that the diffraction patterns of the c-CNF samples closely resembled those of native nanocellulose, displaying characteristic diffraction peaks at approximately 16.5°, 22.5°, and 34.6°, which are indicative of the typical cellulose I structure [26], as shown in Figure 5. The CrI values are presented in Table 1. Our study revealed that the CrI of u-CNFs was at 28.74%, whereas c-CNFs exhibited an increase to 41.03% due to the elimination of non-cellulosic content and extractives from the amorphous regions after TEMPO-mediated oxidation [40]. Our results are consistent with previous studies. For example, Madivoli et al. isolated CNFs from Oryza sativa residues using TEMPO-mediated oxidation and reported similar XRD patterns. They estimated the %CrI as around 42% [40].

### 3.4. Preparation and Morphological Characteristics of c-CNF Hydrogels

In the present study, we developed 14 different formulations of c-CNF hydrogels loaded with PHMB through physical crosslinking for antimicrobial applications. By the optimization of polymer ratios, we successfully created crosslinked hydrogels that were flexible. The homogeneous and translucent characteristics of the 6 and 7% *w*/*v* c-CNFs hydrogel formulations are shown in Figure S1. In contrast, formulations with a c-CNF content lower than 6% *w*/*v* displayed rough surfaces and irregular shapes, indicating incomplete hydrogel formation (Figure S1). The interaction between the amine group in PHMB and the carboxyl of c-CNFs results in the formation of ionic interactions. The amine group in PHMB also interacts with the hydroxyl groups of c-CNFs, facilitating the formation of hydrogen bonds. Additionally, van der Waals forces are present between the C-N, C-O-H or C-O-C interactions. These interactions contribute to the presence of crosslinked junctions within the hydrogel structure and enable the dispersion of PHMB within the continuous c-CNF phase [15,41]. This study identified the optimal amount of c-CNFs as a critical factor influencing the hydrogel structure, thus focusing on 6 and 7% *w*/*v* c-CNF/PHMB hydrogels.

**Figure 6:**
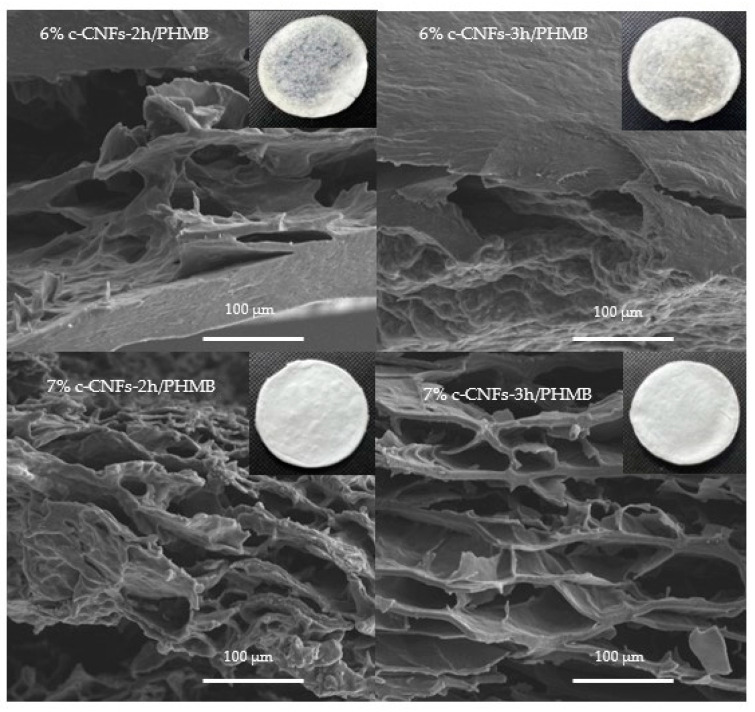
The morphology of the freeze-dried 6% and 7% c-CNF hydrogels loaded with PHMB with variations in the reaction time (2 and 3 h) visualized by FESEM micrographs (cross-sections) at 1000×.

Figure 6 shows the influence of hydrogel composition on the observed structures in the cross-sectional SEM micrographs of 6% and 7% *w*/*v* c-CNF hydrogels. The absence of a separation phase indicates the successful integration of the c-CNFs with PHMB. A notable disparity in the morphology of the polymeric structure was observed when comparing the morphology of the 6% *w*/*v* c-CNF hydrogels to the 7% *w*/*v* c-CNF hydrogels. The 6% *w*/*v* c-CNF hydrogels exhibited lower porosity in the polymer microstructures, while the micrographs of the 7% *w*/*v* c-CNF hydrogels exhibited a highly porous morphology. However, there were no significant differences in the microstructures observed between formulations exhibiting the same c-CNF contents with different reaction times. Furthermore, Liang et al. developed porous PHMB-loaded silk fibroin sponges with antibacterial activity. They found that increasing the mass ratio of PHMB/silk fibroin to 5/100 and 10/100 resulted in a higher number of microscale holes appearing within the pore walls, thereby enhancing connectivity between the pores [15]. Hence, the quantity of added c-CNFs served as a significant parameter determining the morphology and porosity.

### 3.5. Fourier Transform Infrared Spectroscopy (FTIR) of c-CNF Hydrogels Loading PHMB

**Figure 7:**
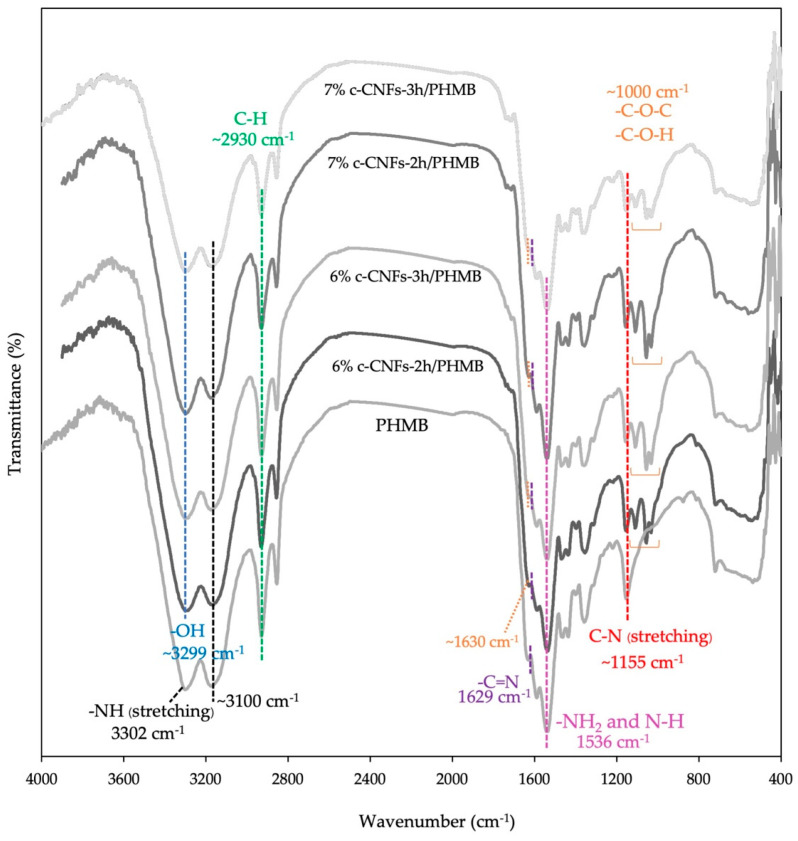
FTIR spectra of PHMB and c-CNF hydrogels loaded with PHMB in the variation of reaction time (2 and 3 h) by TEMPO—mediated oxidation and c-CNF content.

FTIR analysis was conducted to reveal indications of chemical interactions between different composite hydrogels, which can be observed through shifts in chemical signals or changes in intensity. Figure 7 demonstrates the FTIR spectra of PHMB and c-CNF hydrogel formulations. The structural characteristics of PHMB can be identified by analyzing four prominent bands at 3300 cm^−1^, 2930 cm^−1^, 1629 cm^−1^, and 1536 cm^−1^, corresponding to the stretching vibration of N-H, aliphatic C-H, C=N stretching, and amine (-NH_2_) bending vibration, respectively [27,42]. The spectra clearly show peaks at approximately 3300 cm^−1^ and 3100 cm^−1^, corresponding to the stretching vibration of hydroxyl and secondary amine groups, indicating the presence of hydrogen bonds between c-CNFs and PHMB in the hydrogel formulations [43]. Furthermore, the c-CNF/PHMB hydrogels exhibit a distinct absorption peak at 1536 cm^−1^, corresponding to the N–H bending vibration. Additionally, there is an absorption peak at 1155 cm^−1^, representing the C–N stretching vibration. Moreover, the absorption peak around 1000 cm^−1^ corresponds to the stretching vibration of C–O in C–O–H and C–O in C–O–C [15,44,45]. These peaks indicate the presence of van der Waals forces [15]. In particular, a slight shift in the peak position from 1600 cm^−1^ to 1630 cm^−1^ indicates the occurrence of interactions with and adsorption of PHMB in the c-CNF hydrogels via the amine group of PHMB and the carboxylic group of c-CNFs [12]. FTIR analysis provides confirmation of the successful integration of PHMB into the c-CNF matrix.

### 3.6. Puncture Strength, Gel Content, Maximum Swelling Degree, and Drug Content of c-CNF Hydrogel Loaded with PHMB

**Figure 8:**
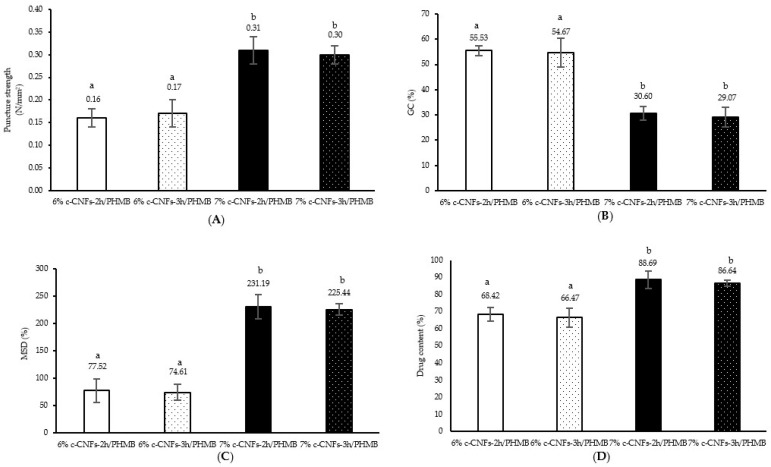
(**A**) Puncture strength, (**B**) gel content (GC), (**C**) maximum swelling degree (MSD), and (**D**) drug content of c-CNF hydrogels containing PHMB. For each test, average values with the same letter are not significantly different. Thus, average values with different letters, e.g., ‘a’ or ‘b’, are statistically different (*p* < 0.05).

An ideal hydrogel for wound dressings should possess a suitable puncture strength, allowing it to maintain its structure and durability throughout the application period. In order to assess the mechanical properties of c-CNF hydrogels loaded with PHMB, the puncture strength was measured to determine the influence of the polymer component. The results are presented in Figure 8 and Table S1. Our results indicated that formulations with a higher CNF content (7% c-CNFs-2 h/PHMB and 7% c-CNFs-3 h/PHMB) exhibited greater puncture strength (~0.3 N/mm^2^) compared to formulations with a lower CNF content (6% c-CNFs-2 h/PHMB and 6% c-CNFs-3 h/PHMB). There was no significant difference in puncture strength between formulations in the same c-CNF content with different reaction times (*p* > 0.05). This observation may be attributed to the presence of a higher number of crosslinked junctions and interactions within the polymeric matrix of 7% c-CNF hydrogels, facilitating the formation of ionic interactions and hydrogen bonds between the amine group in PHMB and the carboxyl and hydroxyl groups of c-CNFs in comparison to 6% c-CNF hydrogels. Our results are consistent with previous studies. Yuan Y. et al. developed a biodegradable starch-based antibacterial film that incorporated nanocellulose and PHMB. They observed an increase in the tensile strength value from 0.76 MPa to 3.44 MPa as the amount of nanocellulose increased. Therefore, the addition of nanocellulose had the potential to improve the mechanical properties of the starch film [45].

The structural stability of PHMB-incorporated c-CNF hydrogels was assessed by measuring the gel content (%GC) in phosphate-buffered saline (PBS) at pH 7.4 at 37 ± 0.5 °C, and the results were presented in Figure 8 and Table S1. The analysis of the gel fraction revealed that the 6% c-CNFs-2 h/PHMB and 6% c-CNFs-3 h/PHMB formulations exhibited a higher %GC (approximately 55%) when immersed in PBS. On the other hand, the formulations containing 7% c-CNFs-2 h/PHMB and 7% c-CNFs-3 h/PHMB, which had higher CNF contents, exhibited a lower %GC (approximately 30%) due to the presence of a highly porous network and the interaction of hydrogen bonds with the immersion media. The polymer matrices contained a significant number of hydrophilic groups (–OH or –COOH), and the higher concentration of these hydrophilic groups increased the polarity and water solubility, thereby resulting in a decrease in gel content [46,47].

The evaluation of the hydrogel’s swelling behavior was examined by immersing it in PBS at pH 7.4 for 24 h, as shown in Figure 8 and Table S1. Our result revealed that the 7% c-CNFs-2 h/PHMB and 7% c-CNFs-3 h/PHMB formulations exhibited an increased %MSD (approximately 225% to 230%) due to their highly porous network and interactions with water through hydrogen bonding. Conversely, the %MSD of the 6% c-CNFs-2 h/PHMB and 6% c-CNFs-3 h/PHMB formulations, which contained lower CNF contents, demonstrated the lowest %MSD in PBS (approximately 75% to 78%). This can be attributed to the reduced presence of hydrogen bonds in their network, resulting in decreased water permeability. The degree of swelling is influenced by the porosity and hydrophilicity of the hydrogel, as water permeates the structure and interacts with the hydrophilic polymers through hydrogen bonding [48]. The composite hydrogels exhibit a porous surface, allowing for the efficient absorption of exudate. They possess a high capacity for water absorption and effective water retention, making them suitable for wound dressing applications [45].

The PHMB content of the 6% c-CNFs-2 h/PHMB, 6% c-CNFs-3 h/PHMB, 7% c-CNFs-2 h/PHMB, and 7% c-CNFs-3 h/PHMB preparations was determined and presented in Figure 8 and Table S1. The drug content of the 7% c-CNFs-2 h/PHMB and 7% c-CNFs-3 h/PHMB hydrogels exceeded 80%, demonstrating significantly higher drug contents compared to the 6% c-CNFs-2 h/PHMB and 6% c-CNFs-3 h/PHMB formulations. This difference may be attributed to the greater porosity observed in the hydrogel structure (Figure 6). High porosity facilitates a higher drug-loading capacity within the hydrogel matrix [49]. Theoretically, ionic interactions and hydrogen bonding could be formed between the amine group in PHMB and the carboxyl and hydroxyl groups of c-CNFs.

Based on our study, the 7% c-CNFs-2 h/PHMB formulation was selected to evaluate its in vitro drug release profile and antibacterial activity. This formulation offers advantages over the 7% c-CNFs-3 h/PHMB formulation as it requires less time and fewer chemicals for surface modification using the TEMPO/NaBr/NaClO system than the 7% c-CNFs-3 h/PHMB formulation, resulting in cost savings. Furthermore, the 7% c-CNFs-2 h/PHMB formulation exhibited desirable characteristics, including a regular shape, high porosity, good mechanical properties, suitable gel content, a good maximum swelling degree, and PHMB contents that were greater than 80%.

### 3.7. In Vitro Drug Release Profile of c-CNFs Hydrogel Loading PHMB

**Figure 9:**
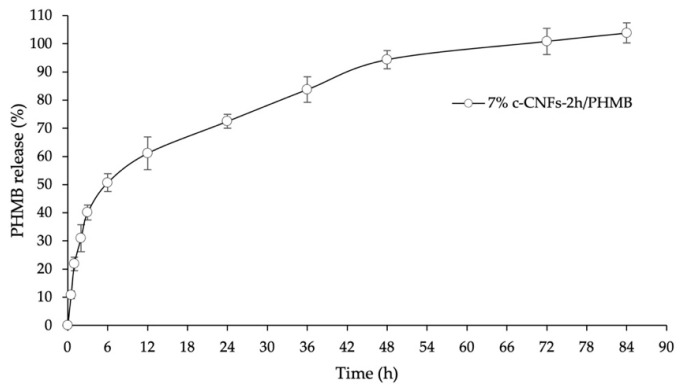
PHMB release profile of 7% c-CNFs-2 h/PHMB hydrogel in PBS pH 7.4.

The drug release profile of the 7% c-CNFs-2 h/PHMB hydrogel was evaluated in phosphate-buffered saline (PBS) at pH 7.4, as shown in Figure 9. This hydrogel exhibited a prolonged drug release pattern. Initially, there was a burst release of PHMB from the c-CNF-loaded hydrogel within the first 4 h, followed by a gradual release reaching approximately 100% within 72 h. This initial burst release can be attributed to the weak bonding between c-CNFs and PHMB, which relies on physical forces such as van der Waals’ forces, hydrogen bonding, and electrostatic adsorption [50]. The subsequent slow release can be attributed to the disruption of the weak physical forces between c-CNFs and PHMB, as well as overcoming diffusion barriers within the surrounding networks [15]. The penetration of the drug within the matrix systems is influenced by the hydrophilicity of the formulation that contains 7% c-CNFs [11,51]. In another study, porous PHMB-loaded silk fibroin sponges with antibacterial activity exhibited burst release within the first 12 h, followed by a slower release for up to 20 days. This behavior was attributed to the presence of a hydrophobic region in the polymer, hindering solvent molecule permeation and reducing drug diffusion from the polymeric matrices [15]. In addition, it could be explained by the crystalline structure of the c-CNFs. To clarify, crystalline structures are usually soluble in water and thus might prevent the permeation of water molecules into the hydrogel’s structure to dissolve PHMB.

### 3.8. Antibacterial Activity of c-CNF Hydrogel Loaded with PHMB

The c-CNF hydrogel loaded with PHMB was assessed for its antibacterial activity against *S. aureus* and *P. aeruginosa* using the disk diffusion method. As a negative control, 7% c-CNFs-2 h film without PHMB was used, while a Whatman disc loaded with 0.3% PHMB served as the positive control. The results demonstrated that the antibacterial activity of 7% c-CNFs-2 h/PHMB against *S. aureus* and *P. aeruginosa* significantly increased compared to the positive control, as shown in Table 2. The porous structure of the hydrogel facilitated increased interactions at the interface between the loaded PHMB and the bacteria, thereby enhancing its antibacterial efficacy. Our results are consistent with previous studies. For example, Ye Y. et al. developed a poly(ethylene glycol) methyl ether acrylate (PEGMA) hydrogel with improved antibacterial activity. They found that the porous structure provided space for bacterial growth, while the extensively exposed surface enhanced bacterial interactions [52].

## 4. Conclusions

The surface modification of CNFs using the TEMPO/NaBr/NaClO system was successful in improving their hydrophilicity. The yield percentages were reasonable and sufficient for the fabrication of c-CNF hydrogels loaded with PHMB through physical crosslinking for antimicrobial applications. Firstly, the potential of carboxylated cellulose nanofibers was confirmed by FESEM, FTIR, DO, CC, and XRD studies. Our study selected c-CNFs-2 h and c-CNFs-3 h as suitable samples based on all the parameters evaluated. Subsequently, c-CNF hydrogels loaded with PHMB were fabricated using physical crosslinking. Among the 14 c-CNF formulations, 7% c-CNFs-2 h/PHMB and 7% c-CNFs-3 h/PHMB exhibited desirable characteristics, such as regular shape, high porosity, good mechanical properties, suitable gel content, and a good maximum swelling degree. The successful integration of PHMB into the c-CNF matrix was confirmed by an FTIR study. Additionally, 7% c-CNFs-2 h/PHMB and 7% c-CNFs-3 h/PHMB were successfully prepared, with PHMB contents exceeding 80%. Consequently, we selected to evaluate the in vitro drug release profile and antibacterial activity of the 7% c-CNFs-2 h/PHMB formulation. This formulation demonstrated a prolonged drug release pattern, with the PHMB release observed over a period of 3 days. Moreover, it exhibited significant antibacterial activity against *S. aureus* and *P. aeruginosa*. Based on the present study, we have concluded that c-CNFs-2 h is the optimized time for surface modification using the TEMPO/NaBr/NaClO system, which can be utilized for the synthesis of carboxylated cellulose nanofibers. Furthermore, our novel approach of synthesizing c-CNF hydrogels loaded with PHMB through physical crosslinking shows promise as a potential system for prolonged drug release in transdermal drug delivery and exhibits excellent antibacterial activity. However, further studies are required to evaluate the in vivo treatment efficacy of the developed c-CNF hydrogels for skin infections.

## Figures and Tables

**Table 1:** Degree of oxidation (DO), carboxylic acid content (CC), and crystallinity index (CrI) of u-CNFs and c-CNFs.

Sample	DO	CC (mmol/g)	CrI (%)
u-CNFs	-	-	28.74 ^a^
c-CNFs-**1 h**	0.0691 ± 0.0027 ^a^	0.4200 ± 0.0163 ^a^	32.48 ^b^
c-CNFs-**2 h**	0.0993 ± 0.0028 ^b^	0.6000 ± 0.0163 ^b^	40.76 ^c^
c-CNFs-**3 h**	0.0971 ± 0.0016 ^b^	0.5867 ± 0.0094 ^b^	41.03 ^c^

For each test, average values with the same letter are not significantly different. Thus, average values with different letters, e.g., ‘^a^’ or ‘^b^’, are statistically different (*p* < 0.05).

**Table 2:** Antibacterial activity of c-CNF hydrogel against *S. aureus* and *P. aeruginosa*.

Samples	Zone of Inhibition (mm)			
	*S. aureus*		*P. aeruginosa*	
7% c-CNFs-2 h	ND	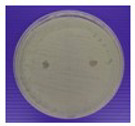	ND	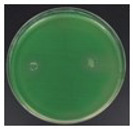
7% c-CNFs-2 h/PHMB	18.90 ± 0.37 ^a^	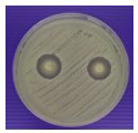	12.47 ± 1.68 ^a^	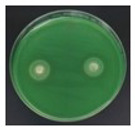
0.3% PHMB	10.91 ± 0.27 ^b^	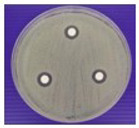	8.08 ± 0.91 ^b^	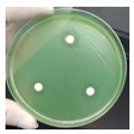

ND means not detected; superscripts with different letters in each column denote statistically different (*p* < 0.05) mean values.

## Data Availability

The data presented in this study are available on request from the corresponding author.

## Supplementary Materials

The following supporting information can be downloaded at https://www.mdpi.com/article/10.3390/polym15173572/s1, Figure S1: The appearance of c-CNF hydrogels loaded with PHMB by the variation of reaction time (2 and 3 h) and the ratio of c-CNFs (1–7% *w*/*v*) and Table S1: Puncture strength, gel content (GC), maximum swelling degree (MSD), and drug content of c-CNF hydrogels containing PHMB.
