# Supplementary material for: Surface-Modified Carboxylated Cellulose Nanofiber Hydrogels for Prolonged Release of Polyhexamethylene Biguanide Hydrochloride (PHMB) for Antimicrobial Applications
Source: Polymers (Basel). 2023 Aug 28;15(17):3572. doi: 10.3390/polym15173572 (PMC10490332; doi:10.3390/polym15173572)
Supplement: Supplementary file 1 [file polymers-15-03572-s001.zip › polymers-2506612-supplementary.pdf]

polymers

Supplementary Materials: Surface-Modified Carboxylated Cellulose Nanofiber Hydrogels for Prolonged Release of Polyhexamethylene Biguanide Hydrochloride (PHMB) for Antimicrobial Applications

Pichapar O-chongpian[1], Tanpong Chaiwarit[1], Kittisak Jantanasakulwong[2,3], Pornchai Rachtanapun[2,3], Patnarin Worajittiphon[4], Nutthapong Kantrong[5], and Pensak Jantrawut[1,3*]

[1] Department of Pharmaceutical Sciences, Faculty of Pharmacy, Chiang Mai University, Chiang Mai 50200, Thailand; pichaparo@gmail.com (P.O.); tanpong.c@gmail.com (T.C.); pensak.amuamu@gmail.com (P.J.)
[2] Division of Packaging Technology, Faculty of Agro-Industry, Chiang Mai University, Chiang Mai 50100, Thailand; kittisak.jan@cmu.ac.th (K.J.); pornchai.r@cmu.ac.th (P.R.)
[3] Center of Excellence in Agro Bio-Circular-Green Industry (Agro BCG), Chiang Mai University, Chiang Mai 50200, Thailand
[4] Department of Chemistry, Faculty of Science, Chiang Mai University, Chiang Mai 50200, Thailand; patnarin.w@cmu.ac.th
[5] School of Dentistry, Mae Fah Luang University, Chiang Rai 57100, Thailand nutthapong.kan@mfu.ac.th
* Correspondence: pensak.amuamu@gmail.com, pensak.j@cmu.ac.th; Tel.: +6653944309

Citation: O-chongpian, P.; Chaiwarit, T.; Jantanasakulwong, K.; Rachtanapun, P.; Worajittiphon, P.; Kantrong, N.; Jantrawut, P. Supplementary Materials: Surface-Modified Carboxylated Cellulose Nanofiber Hydrogels for Prolonged Release of Polyhexamethylene Biguanide Hydrochloride (PHMB) for Antimicrobial Applications. *Polymers* **2023**, *15*, 3572. https://doi.org/10.3390/polym15173572

Academic Editor: Jem-Kun Chen

Received: 30 June 2023
Revised: 23 August 2023
Accepted: 25 August 2023
Published: 28 August 2023

Copyright: © 2023 by the authors. Licensee MDPI, Basel, Switzerland. This article is an open access article distributed under the terms and conditions of the Creative Commons Attribution (CC BY) license (http://creativecommons.org/licenses/by/4.0/).

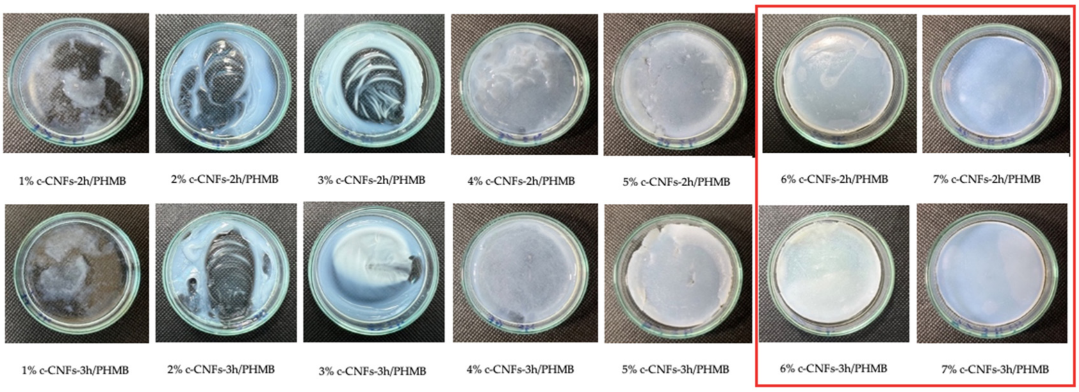

Figure S1. The appearance of c-CNFs hydrogels loading PHMB by the variation of reaction time (2 and 3 h.) and ratio of c-CNFs (1-7% w/v).

Table S1. Puncture strength, gel content (GC), maximum swelling degree (MSD) and drug content of c-CNFs hydrogels containing PHMB.

**Formulations**	**Puncture strength)N/mm^2(**	**GC (%)**	**MSD (%)**	**Drug content (%)**
6% c-CNFs-2h/PHMB	0.16 ± 0.02^{a}	55.53 ± 1.97^{a}	77.52 ± 21.37^{a}	68.42 ± 3.93^{a}
6% c-CNFs-3h/PHMB	0.17 ± 0.03^{a}	54.67 ± 5.57^{a}	74.61 ± 14.04^{a}	66.47 ± 5.71^{a}
7% c-CNFs-2h/PHMB	0.31 ± 0.03^{b}	30.60 ± 2.71^{b}	231.19 ± 22.09^{b}	88.69 ± 4.99^{b}
7% c-CNFs-3h/PHMB	0.30 ± 0.02^{b}	29.07 ± 3.91^{b}	225.44 ± 10.32^{b}	86.64 ± 1.90^{b}

For each test, average values with the same letter are not significantly different. Thus, average values with the different letter, e.g., ‘a’ or ‘b’ are statistically different ($p < 0.05$).
